# The significance of elevated plasma expression of microRNA 106b~25 clusters in gastric cancer

**DOI:** 10.1371/journal.pone.0178427

**Published:** 2017-05-31

**Authors:** Fangxuan Li, Yuenan Guo, Juntian Liu, Rupeng Zhang

**Affiliations:** 1 Department of Cancer Prevention Center, Tianjin Medical University Cancer Institute and Hospital, Tianjin, China; 2 National Clinical Research Center for Cancer, Tianjin, China; 3 Key Laboratory of Cancer Prevention and Therapy, Tianjin, China; 4 Tianjin’s Clinical Research Center for Cancer, Tianjin, China; 5 Department of Gastric Cancer Surgery, Tianjin Medical University Cancer Institute and Hospital, Tianjin, China; University of South Alabama Mitchell Cancer Institute, UNITED STATES

## Abstract

**Objective:**

Concentrating on oncogenic role and increased plasma expression of microRNA(miR) 106b~25 clusters (involving miR 106b, miR 93 and miR 25), we evaluated significance of the over-expression of plasma miR 106b~25 in GC.

**Methods:**

Based on 65 pairs matched GC patients and health controls, we explored clinical significance of miR 106b~25 for GC and compared their diagnostic performance with conventional tumor biomarkers including CA724, CA242, CA199 and CEA.

**Results:**

Both miR 106b~25 cluster and conventional tumor biomarkers were significantly elevated in GC (All *P*<0.05). In ROC curves, miR 106b had the highest AUC (0.898) in diagnosing GC with optimal sensitivity of 86.2% and specificity of 92.3% at the cut-off value of 1.385. MiR 25 had moderate diagnostic efficacy (AUC = 0.817) with sensitivity of 87.6% and specificity of 76.9% at the threshold of 1.015. The AUC of miR 93 (0.756) was the lowest. The AUC, sensitivity, accuracy and Youden index of miR 106b were higher than all of four conventional biomarkers, while its specificity is higher than CA242 and CA724. The AUC of miR 25 was also higher than CA724, CA242 and CA199, while AUC of miR 93 was only higher than CA199 and CA724. Compared the diagnostic efficacy via ROC curves, miR 106b was significantly higher diagnostic efficacy than CA724, CA242 and CA199, the diagnostic efficacies of miR 93 and miR 25 were significantly higher than CA199(all *P*<0.05).

**Conclusions:**

Plasma miR 106b~25 cluster, especially miR 106b, were significantly increased in GC patients and may be hopeful diagnostic biomarkers.

## Introduction

Gastric cancer (GC) is the fifth leading cause of cancer and the third leading cause of cancer death making up 7% of cancer cases and 9% of deaths globally [1]. As reported, due to absence of effective screening method for early GC [2], the prognosis of GC is poor with the 5-year survival rate less than 10%. As a heterogeneous and multifactorial disease, GC remains to be a noticeable public health problem in East Asia (Japan, China and Korea) because of its highest morbidity and mortality [3]. Especially in China, a developing country, the proportion of early GC is always at a lower level compared with the other two countries, since gastroscopy is not yet universal for screening in high-risk group [4].

For several decades, gastroscopy has been suggested as the first choice for screening early stage GC; however, due to its invasive character and social economic reasons, it is remaining low compliance in the clinical setting [4]. Therefore, cost-effective and non-invasive biomarkers with high sensitivity and specificity are eagerly needed to enable early detection of GC. For some cancers, blood-based proteins have been demonstrated and widely used as biomarkers in clinical diagnosis, such as AFP for hepatocellular carcinoma [5] and CA125, HE4 for ovarian cancer [6]. Unfortunately, situation is quite perplexed for GC. After decades of research, there is still a lack of effective biomarkers for GC diagnosis.

Common tumor biomarkers, such as CA724, CA242, CA199 and CEA, have exhibited poor diagnostic value in digestive tract malignancies screening, and are commonly used to identify the disease stage and monitor follow-up [7,8]. Elevation of plasma CA724 has been reported in around 40.0% of GC patients in most studies [9,10]. Positive rate of CA242 in GC patients is around 21.2%, while the positive rate of CA199 has been reported to be in 18.0% of GC patients in previous study [11]. The positive rate of preoperative serum CEA in GC patients has been reported to be 19.1%~25.0% [12].

MicroRNAs (miRs), which regulate gene expression at the post-transcriptional level by binding to the untranslated regions (UTRs) of mRNAs, are small non-coding RNAs of 18–22 nucleotides in length [13]. Since their discovery in 1993, emerging evidence showed that expression change of miRs is associated with cancer, involving GCs [14,15]. Because they are on intimate terms with tumor genesis, miRs have attracted great attention from researchers to search sensitive and specific biomarkers for early stage cancer. Since plasma miRs are protected from RNase digestions, they remain stable for a long period of time even under extremely harsh conditions. In addition, the abundance of plasma miRs normally does not vary in different gender [16]. Therefore, researchers suggested that miRs may be an ideal candidate for cancer detection [17].

However, to our knowledge, there are few studies comparing the diagnostic application of plasma miRs with conventional tumor markers in GC. Wu et al accessed miR 421 as a new potential diagnosis biomarker with higher sensitivity and specificity than CEA and CA125 in GC [18]. They also reported that circulating miR 21 serve as a better diagnostic biomarker for GCs compared with CA199 and CEA in 2015 [19].

MiR 106b~25 cluster (composed of the highly conserved miR 106b, miR 93, and miR 25), which negatively regulates transcription factor E2F1 and transforming growth factor-β(TGF-β) pathway, plays an important role in GC [20]. With a plurality of different molecular targets, the miR 106b~25 polycistron exerted potential proliferative, anti-apoptotic, cell cycle promoting effects in vitro and tumorigenic properties in vivo [21, 22]. In our previous studies in vitro, we found that expression of miR 106b, miR 93 and miR 25 was significantly higher in GC cell lines and cancer tissues [23]. What's more, the expressive level of plasma miR 106b~25 cluster was also statistically significant higher than healthy volunteers [24]. We searched differential expression of this cluster in TCGA database, there were 443 cases opened for GC mRNA & miRNA expression involving 445 GC patients and 46 normal volunteers. We analyzed these data and found that, in comparison miR expression of GC and normal cases, the fold change of miR 93 was >1(logFC = 1.117, *P*<0.05), while the fold change of miR 106b was nearly 1(logFC = 0.971) (shown as S1 Table). So, we presumed whether the miR 106b~25 are candidate biomarkers for GC diagnosis. Thus, this study was designed to explore clinical application of miR 106b~25 in GC diagnosis and compare their diagnostic performance with conventional tumor biomarkers CA724, CA242, CA199 and CEA based on 65 pairs matched GC patients and health controls.

## Methods

### Patients

Inclusive criteria of GC patients including: (1) Patients underwent gastrectomy for GC between February 2014 and June 2015 at Tianjin Medical University Cancer Institute and Hospital. (2) Patients with gastric adenocarcinoma identified by histopathologic examination. Exclusive criteria of GC patients including: (1) Patients received chemotherapy or radiotherapy before collecting samples. (2) Patients with chronic disease or infectious diseases. (3) Patients with history of other malignancy.

As a result, we collected pre-operative blood samples from 65 patients with GC, as well as from matched 65 healthy volunteers. All clinicopathological data of GC were assessing according to the 7^th^ GC tumor-node-metastasis (TNM) staging system of the Union of International Control Cancer (UICC).

Informed consent was taken from every subject, and the Human Research Ethics Committee of Tianjin Medical University approved all aspects of this study.

### Samples

Blood samples were obtained immediately following diagnosis and prior to any oncological treatment. The peripheral blood (5 mL) samples were collected into ethylenediaminetetraacetic acid (EDTA) anticoagulative tubes immediately. After collection, the blood samples were subjected for isolation of cell-free nucleic acids by using a three-spin protocol (2,000g for 30 min, 4,000g for 5 min, 8,000g for 5 min) to prevent contamination from cellular nucleic acids. Plasma samples were then stored at -80°C until further processing. Blood samples for conventional tumor marker determination were separated by centrifugation and aliquots were stored at -20°C until assayed.

### RNA extraction and detection of miRs

Plasma RNA was extracted by using acid phenol according to the manufacturer’s instructions. Total RNA was quantified by microfluidics analysis (Gene Quant, Switzerland). The amounts of miRs were quantified in duplicate by quantitative reverse transcription polymerase chain reaction (RT-PCR) using the human TaqMan MicroRNA Assay Kits (Applied Biosystems, Foster City, CA, USA). After the reverse transcription reaction which was carried out with TaqMan MicroRNA Reverse Transcription Kit (Applied Biosystems), cDNA solution was amplified using TaqMan Universal PCR Master MixII with no AmpErase UNG (Applied Biosystems). RT-PCR was run on 7500 Real Time PCR system (Applied Biosystems), and the cycle threshold (Ct) values were calculated with the SDS 1.4 software (Applied Biosystems). All reactions were performed in triplicate.

Analysis of U6 levels in serum of GC patients and healthy controls revealed that the expression of this conserved miRNA remained at comparable levels in serum among all participants in this study (U6 quantification in GC patients and health control: 26.34±1.72 *vs* 27.01±1.40, *P* = 0.199), which indicated that U6 was an appropriate normalization control. Through the 2^-ΔΔCt^ method, expressions of miRs from plasma samples were normalized by U6. The Ct was calculated by subtracting the Ct values of reference substance from the Ct values of the interesting miRs. Mean Ct and standard deviation values were calculated without outliers (i.e., replicates with Ct differing by more than one cycle from the median). The ΔΔCt was then calculated by subtracting ΔCt of the median of control samples from ΔCt of study group. Fold change was calculated by the equation 2^-ΔΔCt^ [25].

### Conventional tumor markers

Specimens were tested by electrochemiluminescence immunoassay according to standard procedure of Roche Company’s kit and Roche E170 automatic immunity analyzer.

### Statistical methods

For statistical methods, The Chi-square and the Fisher’s exact tests were used for categorical variables. The independent t-test and ANOVA was used to analyze the statistical significance of continuous variable. Conditional logistic regression was applied to adjust the matched variables via enter method of binary Logistic regression. ROC curves were to describe and compare the accuracy of diagnostic tests via binormal model. The *P* value of *P*<0.05(two-sided) was regarded as statistically significant. All analyses were performed using SPSS version 17.0. The pair-wise comparison of ROC curves was performed using MEDCALC software based on Hanley&McNeil method. The accuracy = (True positive+true negative)/(True positive+true negative+false positive+false negative). Youden's index = Sensitivity+ Specificity-1. Optimal cut-off value was set as the threshold with the highest Youden's index.

## Results

### The expression of miR 106b~25 cluster and tumor markers in plasma of GC patients and health controls

**Fig 1:**
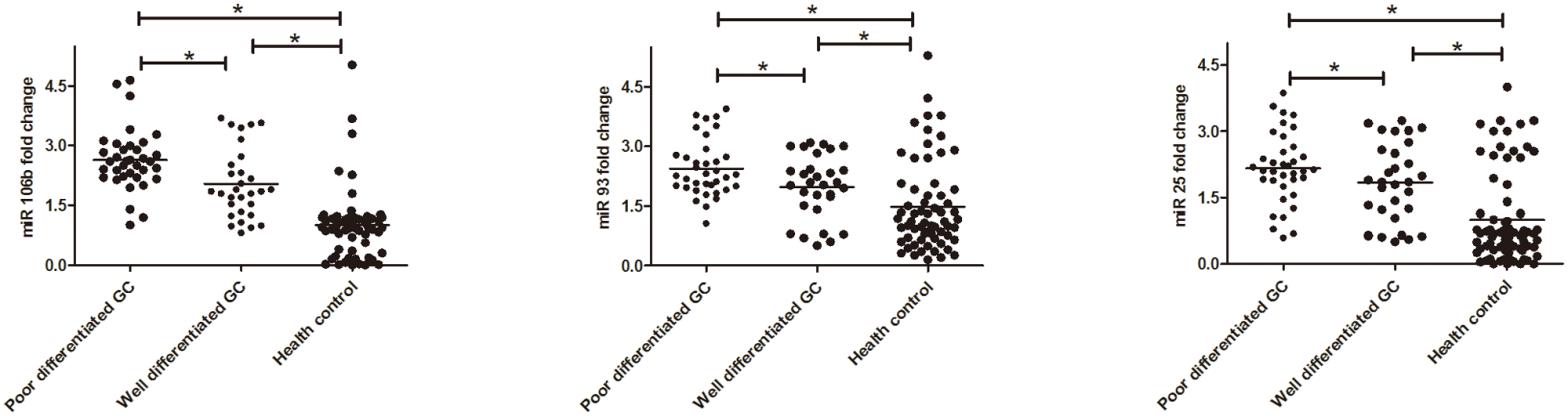
The expression of miR-106b~25 clusters in plasma of GC patients and health controls. (a) miR-106b, (b) miR-93 and (c)miR-25 were significant higher in poor differentiated GC and well differentiated GC than control groups. Additionally, (a) miR-106b, (b) miR-93 and (c)miR-25 expression were significant higher in poor differentiated GC than well differentiated GC. *P<0.05.

**Fig 2:**
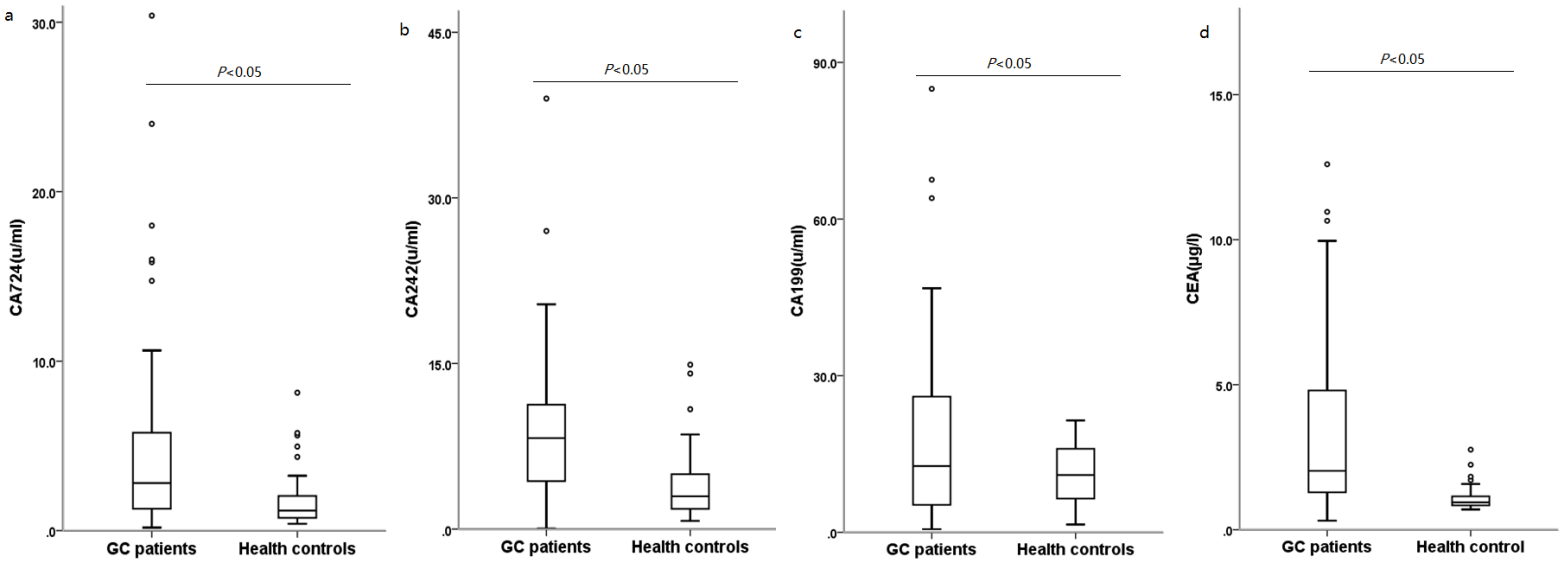
The expression of tumor markers in GC patients and health controls. The concentration of (a)CA-72.4, (b)CA-242, (c)CA-19.9 and (d)CEA were significant higher than healthy controls.

We matched the GC patients with age, sex, main blood biochemistry and blood routine indexes. The baseline characteristics of matched GC patients and health controls were showed in Table 1. We also adjusted these baseline characteristics by conditional logistic regression. Both miR 106b~25 cluster and tumor markers were significantly elevated in GC patients in comparison to healthy controls, as shown in Fig 1 and Table 1. As shown in Fig 1, the fold change of miR 106b was >1 in 61/65 GC patients, the fold change of miR 93 was >1 in 60/65 GC patients, the fold change of miR 25 was >1 in 55/65 GC patients, shown in S2 Table. The expression of miR 106b, miR 93 and miR 25 were significant higher in poor differentiated GC and well differentiated GC than control groups. What's more, miR 106b, miR 93 and miR 25 expression were significant higher in poor differentiated GC patient than well differentiated GC patients. Meanwhile, the concentration of CA724, CA242, CA199 and CEA were significant higher than healthy controls either (all *P*<0.05, Fig 2).

**Table 1:** The baseline characteristics, expression of miR 106b~25 cluster and tumor markers in plasma of GC and health controls.

	GC patients	Health control	*t*	*P*	Logistic regression^#^	
					OR(95% CI)	*p*
Age	54.096±8.46	56.171±8.91	1.362	0.175	*NA*	
Sex(male/female)*	50/15	50/15	0.000	1.000	*NA*	
WBC(×10^9^)	7.01±5.03	7.41±9.48	0.820	0.404	*NA*	
RBC(×10^12^)	4.39±0.741	4.41±0.595	0.168	0.867	*NA*	
Hb(g/L)	130.46±22.28	133.92±25.33	0.826	0.414	*NA*	
ALT (U/L)	15.09±13.65	17.97±19.07	0.989	0.325	*NA*	
AST (U/L)	18.29±10.08	19.36±15.66	0.461	0.646	*NA*	
AlbP(g/L)	44.33±3.85	43.77±7.08	0.561	0.576	*NA*	
TP (g/L)	72.45±6.45	70.44±10.06	1.306	0.194	*NA*	
Cre (μmol/L)	74.41±7.57	75.84±15.29	0.492	0.623	*NA*	
BUN (μmol/L)	6.66±9.85	5.69±1.87	0.772	0.442	*NA*	
miR 106b	2.36±0.86	1.00±0.86	9.029	0.000	7.942 (3.393~16.053)	0.000
miR 93	2.23±0.78	1.48±1.13	4.381	0.000	2.275(1.468~3.527)	0.000
miR 25	2.02±0.85	0.91±1.02	6.727	0.000	3.162(2.126~5.317)	0.000
CA724	6.85±12.28	1.74±1.50	3.352	0.001	1.564(1.215 ~2.013)	0.001
CA242	29.84±83.54	3.85±3.13	2.524	0.014	1.372(1.203~1.567)	0.000
CA199	72.01±191.96	11.06±5.64	2.578	0.012	1.053 (1.011~1.098)	0.013
CEA	7.32±20.02	1.01±0.38	2.558	0.013	18.638(5.347~64.962)	0.000

*For these categorical variables, the *x*^*2*^ value and *P* value were calculated by Chi-square tests. *NA*: not applied.

^#^Conditional logistic regression account for adjusting matching variables.

### The diagnostic value of miR 106b~25 cluster for GC

**Fig 3:**
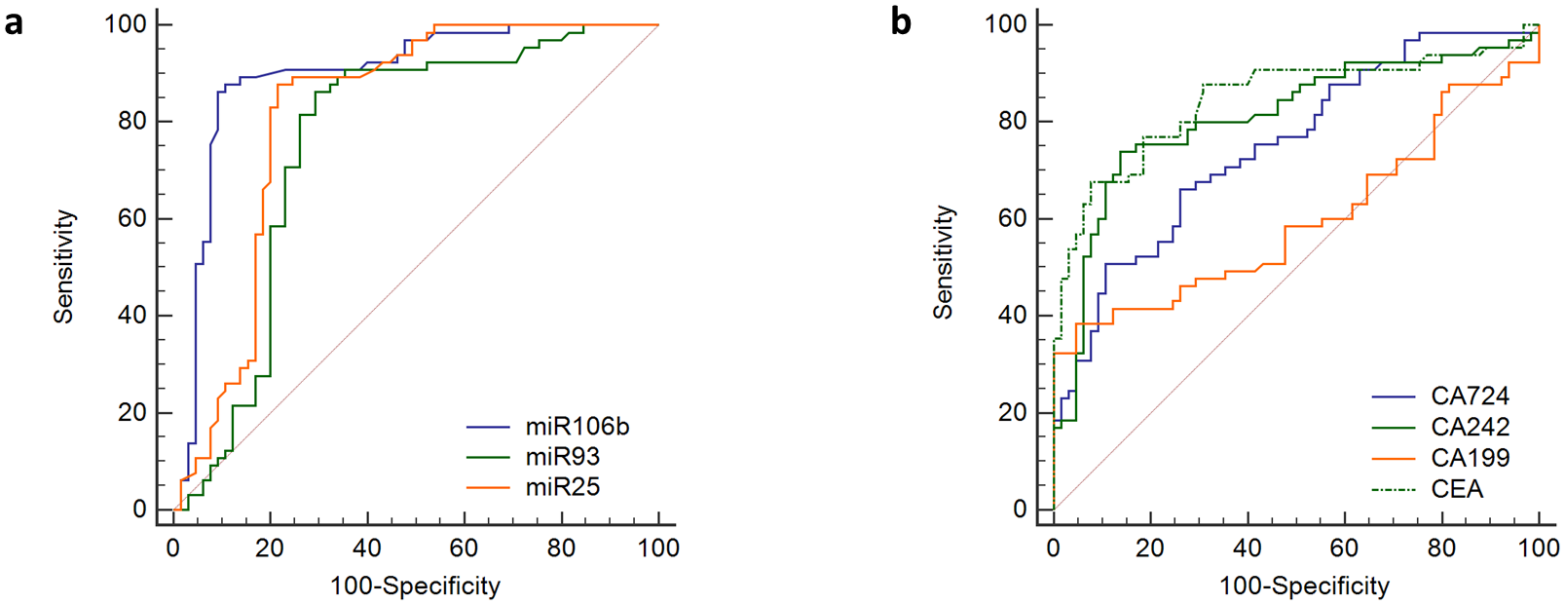
The ROC curves for miR-106b~25 clusters and tumor markers. (a) The AUC of miR-106, miR-25 and miR-93 were 0.898, 0.756 and 0.817 respectively (all *P*<0.05); (b) The AUC of CA724, CA242, CA199 and CEA were 0.751, 0.809, 0.598 and 0.846 respectively(all *P*<0.05).

To determine whether plasma levels of miR 106b, miR 93 and miR 25 have diagnostic values for GC, the ROC curve was applied to analyze their diagnostic sensitivity and specificity (Fig 3 and Table 2). Among them, miR 106b had the highest AUC in distinguishing patients with GC from healthy controls. At the threshold of 1.385, the optimal sensitivity and specificity of miR 106b were 86.2% and 92.3% [area under curve (AUC) = 0.898(0.839–0.958)]. MiR 25 had moderate diagnostic efficacy with the optimal sensitivity of 87.6% and specificity of 76.9% [AUC = 0.817(0.738–0.897)] at the cut-off of 1.015. The AUC of miR 93 was the lowest with the optimal sensitivity of 81.5% and specificity of 73.8% at the threshold of 1.765 [0.756(0.665–0.846)], as shown in Fig 3a.

**Table 2:** The diagnostic value of miR 106b~25 cluster and tumor markers for GC.

	Cut-off	Sensitivity(%)	Specificity(%)	FPR(%)	FNR(%)	Accuracy(%)	Youden index(%)	AUC	*P*
miR 106b	1.385	86.2	92.3	7.7	13.8	89.2	75.5	0.898(0.839–0.958)	0.000
miR 93	1.765	81.5	73.8	26.2	18.5	77.6	53.7	0.756(0.665–0.846)	0.000
miR 25	1.015	87.6	76.9	23.1	12.4	85.3	64.5	0.817(0.738–0.897)	0.000
CA724	2.92	49.2	89.2	10.8	50.8	69.2	39.1	0.751(0.668–0.833)	0.000
CA242	5.68	73.8	86.1	13.9	26.2	80.0	58.7	0.809(0.731–0.887)	0.000
CA199	19.26	32.3	100	0.0	67.7	66.1	33.2	0.598(0.497–0.698)	0.051
CEA	1.40	67.6	92.3	7.7	32.4	80.0	58.9	0.846 (0.775–0.916)	0.000

AUC: Area under curve. Positive were defined as≥cut-off value; Negative were defined as < cut-off value; The sensitivity, specificity, FPR, FNR, accuracy and Youden index were calculated based on the number of positive cases and negative cases. FPR: False positive rate; FNR: False negative rate

In order to determine whether the plasma level of miR 106b, miR 93 and miR 25 have clinical values for GC diagnosis, the FPR (False positive rate) and FNR(False negative rate) and diagnosis efficiency were calculated. As shown in Table 2, the results indicate that three components of miR 106b~25 cluster, especially the miR 106b had lower FPR and FNR, which indicated that miR 106b~25 cluster, especially the miR 106b, have high diagnosis values for GC.

### The diagnostic performance of miR 106b~25 cluster in comparing with tumor markers

The diagnostic value of circulating miR 106b~25 was evaluated in comparison to conventional tumor biomarkers CA724, CA242, CA199 and CEA. The AUC, sensitivity, accuracy and Youden index of miR 106b was higher than all four conventional biomarkers, while the specificity is higher than CA242 and CA724. The AUC of miR 25 was also higher than CA724, CA242 and CA199 (Fig 3b), while AUC of miR 93 was only higher than CA199 and CA724. miR 93 and miR 25 had higher sensitivity but lower specificity in comparing with all conventional biomarkers. In Table 3, we directly compared the ROC curves of circulating miR 106b~25 with four conventional tumor markers, the diagnostic efficacy of miR 106b were significantly higher than CA724, CA242 and CA199; the diagnostic efficacies of miR 93 and miR 25 were significantly higher than CA199(all *P*<0.05, Table 3). The diagnostic performance of miR 106b~25 cluster in distinguishing patients with GC from healthy controls was showed in Table 4.

**Table 3:** The comparison of ROC curves among miR 106b~25 and tumor markers.

ROC curves comparison	CA724		CA242		CA199		CEA	
	*z*	*P*	*z*	*P*	*z*	*P*	*z*	*P*
miR 106b	2.913	0.004	1.794	0.044	4.889	0.001	1.184	0.236
miR 93	0.080	0.936	0.963	0.335	2.425	0.015	1.590	0.111
miR 25	1.084	0.278	0.137	0.891	3.447	0.006	0.538	0.597

**Table 4:** The diagnostic performance of miR 106b~25 cluster and tumor markers for GC according to cut-off value by ROC curves.

Index(*n*)		Total	GC (*n* = 65)	Health controls (*n* = 65)	*x*^*2*^	*P*
miR 106b	Positive	61	56	5	80.335	0.000
	Negative	69	9	60		
miR 93	Positive	70	53	17	40.114	0.000
	Negative	60	12	48		
miR 25	Positive	68	57	11	65.247	0.000
	Negative	62	8	54		
CA724	Positive	91	32	7	22.894	0.000
	Negative	39	22	58		
CA242	Positive	57	48	9	47.520	0.000
	Negative	73	17	56		
CA199	Positive	21	21	0	25.046	0.000
	Negative	109	44	65		
CEA	Positive	49	44	5	49.819	0.000
	Negative	81	21	60		

Positive were defined as≥cut-off value; Negative were defined as < cut-off value. The cut-off value was refer to Table 2. The diagnostic performance of miR 106b~25 cluster were calculated based on the number of positive cases and negative cases.

### The association between miR 106b~25 cluster and clinicopathological features of GC patients

Then, we analyzed the association between miR 106b~25 cluster and clinicopathological features of GC patients according to cut-off value of ROC curves. Table 5 showed that the positive expression of miR 106b was related with tumor size, Borrmann type, histological grade, depth of invasion(T1-T3/T4), lymph node metastasis(N0/N1-N3) and TNM stage (all *P*<0.05). The positive expression of miR 93 was associated with tumor size, depth of invasion(T1-T3/T4), TNM stage (all *P*<0.05). The positive expression of miR 25 was related to tumor size, lymph node metastasis and TNM stage (all *P*<0.05).

**Table 5:** The association between miR 106b~25 cluster and clinicopathological features of GC.

Clinicopathological Features	*n*	miR 106b		*x*^*2*^	*p*	miR 93		*x*^*2*^	*p*	miR 25		*x*^*2*^	*p*
		Positive (*n* = 56)	Negative (*n* = 9)			Positive (*n* = 53)	Negative (*n* = 12)			Positive (*n* = 57)	Negative (*n* = 8)		
Age(year) *	65	54.6±8.7	55.7±8.8	0.319	0.750	57.9±8.0	53.3±10.8	1.523	0.132	57.1±8.9	60.0±9.13	0.904	0.369
Sex				2.687	0.194			0.341	0.717			2.737	0.182
male	50	45	5			40	10			42	8		
female	15	11	4			13	2			15	0		
Tumor size				13.804	0.000			9.726	0.003			12.055	0.001
<5	28	19	9			18	10			20	8		
≥5	37	37	0			35	2			37	0		
Borrmann type				15.670	0.000			4.361	0.052			1.924	0.250
I+ II	26	17	9			18	8			21	5		
III+ IV	39	39	0			35	4			36	3		
Histological type				1.658	0.264			0.127	0.721			0.051	0.822
Intestinal	46	38	8			37	9			40	6		
Diffuse	19	18	1			16	3			17	2		
Histological grade				4.649	0.006			2.896	0.114			1.181	0.450
Well differentiated	29	22	7			21	8			24	5		
Poor differentiated	36	34	2			32	4			33	3		
T stage				8.211	0.004			6.299	0.019			2.934	0.119
T1-3	23	16	7			15	8			18	5		
T4	42	40	2			38	4			39	3		
N stage				6.115	0.021			2.593	0.184			5.340	0.022
N0	41	32	9			31	10			33	8		
N1-3	24	24	0			22	2			24	0		
TNM stage				4.649	0.031			5.499	0.026			11.325	0.001
I+II	29	22	7			20	9			21	8		
III+IV	36	34	2			33	3			36	0		

Positive were defined as≥cut-off value; Negative were defined as < cut-off value. The cut-off value was referring to Table 2. The comparison between the GC group and Health control were calculated based on the number of positive cases and negative cases.

*Continuous variable was described by *Mean*±*Standard Deviation* and compared with *independent sample t test*.

## Discussion

In cancer screening and early diagnosis, diagnostic tests through noninvasive means are preferable, therefore, peripheral blood-based test can be easily and widely accepted. Several studies have focused on the role of various biomarkers in early stage cancer progression monitoring and diagnosis. However, the tumor markers, such as CA724, CA242, CA199 and CEA, are not reliable independent markers for early stage cancer screening or diagnosing, as their levels also increase in certain non-neoplastic diseases, including ulcerative colitis, pancreatitis, cirrhosis and hypothyroidism [26]. In this study, although the specificity was high, sensitivity of these four tumor markers were relatively low, ranged from 30% to 72%, accuracy were around less than 80%. Hence, there remains be a high demand for novel and reliable biomarkers with high sensitivity and specificity.

Circulating miRs as novel tumor biomarkers have been demonstrated promising results in preclinical research and given great hope as biomarkers for future cancer early diagnosis. Recently, many articles further revealed the potential of miRs in the early detection and prognosis in several malignancies, such as lung cancer [27], colorectal cancer [28] and early breast cancer. The GC miRNA expression profile has been studied by different methodologies and some miRNAs [14]. Notably, circuiting miR 21, miR 20a, miR 221, miR 378 and miR 421 have been reported to be potential biomarkers for tumor diagnosis and prognosis. In previous study for GC, The AUC value of miR-21 in serum was 0.912, with the sensitivity of 88.4% and the specificity of 79.6% [19]. The sensitivity and specificity of miR 421 were 90.0% and 85.7%, with the AUC of 0.779 [18]. The AUC using miR 20a, and miR 221 were 0.8593 and 0.7960, respectively. miR 378 exhibited a high diagnostic value, with an AUC of 0.861, sensitivity of 87.5% and a specificity of 70.7% [14]. Concentrating on the miR 106b~25, our study showed that AUC, sensitivity and specificity of miR 106b were higher than miR 421, miR 20a, miR 221 and miR 378. In a large-scale analysis [14], the AUC of miRNA106b was the greatest compared with the other miRs (AUC = 0.721), which was in line with our study. In Cai H et al's research, the AUC using miR-106b was 0.7733 [29]. Compared with previous reports, the AUC, sensitivity, and specificity of miR 25 are also higher than miR 378 in our study.

MiRs had the advantage of high stability at room temperature, low degradation in multiple freezing thawing processes and high specificity of tissue distribution [30,31]. Thus, to protect samples from degradation, quantification of miRs may require stabilizing miRs sample and shortening testing time. In our study, to minimize miR degradation, samples were immediately processed for miR extraction after blood collection and stored at -80°C before PCR analysis. The expression of some miRs is specific to tissues or biological stages, and their level can be easily detected by various methods.

Accumulating data has showed that miR 106b~25 cluster plays oncogenic roles in cancers. This cluster has been reported to be up-regulated in several cancers, including multiple myeloma, esophageal squamous cell carcinoma, pancreatic cancer, hepatocellular carcinoma et al. [32,33]. The trigger targets of this oncogenic process are E2F1 and TGF-β [21], while other targets just like Retinoblastoma protein (RB) gene [32] and phosphatase and tensin homolog deleted on chromosome (PTEN) have been demonstrated in mechanism of miR 106b~25 [33]. All of these targets play crucial role as an intrinsic factor of gastric carcinogenesis. In our previous studies, we had demonstrated expression of miR 106b, miR 93 and miR 25 were significantly higher in GC cell lines [23], tumor tissues and plasma form GC patients [24]. As new biomarkers, it still needs further exploration to verify that whether these miRs have advanced sensitivity and specificity compared to traditional tumor markers. In this study, we evaluated the optimal cut-off point of for miR 106b miR 93, and miR 25 diagnosis GC via ROC curve. In comparing with conventional tumor biomarkers, all these three miRs were performed well in diagnosis of malignance. Diagnostic efficacy of miR 106b were significantly higher than CA724, CA242 and CA199; the diagnostic efficacies of miR 93 and miR 25 were significantly higher than CA199. To improve the early diagnosis of GC via miR 106b~25, more rigorously, early GC should be sub-analyzed. However, in China, the GC are often detected at advanced stage, so our data included few early GC patients. Thus, in further, multi-center and larger scale studies were needed to verify the clinical diagnostic accuracy in early GC.

It was reported that miR 106b~25 were also evaluated in some non-neoplastic diseases such as type 2 diabetes mellitus, atherosclerosis, ulcerative colitis etc [20]. In our study, patients with chronic disease or infectious diseases and other malignances were excluded to focus on the correlation between miR 106b~25, conventional biomarkers and GC. However, in clinic, if patients had other co-morbidities or disease mentioned above, the expression level of miR 106b~25 will be affected. So, large-scale analysis from all GC patients were needed to verify validity of miR 06b~25 for clinic diagnosis.

Concentrating on clinicopathological features of GC, miR 106b~25 were also correlated with disease stage. The positive expression of miR 106b was related with tumor size, Borrmann type, histological grade, depth of invasion, lymph node metastasis and TNM stage. The positive expression of miR 93 was associated with depth of invasion. The positive expression of miR 25 was related to tumor size and TNM stage.

In conclusion, based on miR 106b~25 qRT-PCR quantification method, our study indicated that circulating plasma miR 106b~25 cluster, especially miR 106b, was significantly increased in GC patients and could serve as GC diagnostic biomarkers. What’s more, higher expression of miR 106b~25 cluster were correlated with clinicopathological features of GC patients.

## Supporting information

S1 Table Differential expression of miR-106b~25 cluster in GC patients and normal volunteers from TCGA database. (XLSX)

S2 Table The baseline characteristics, expression of miR 106b~25 cluster and tumor markers in plasma of 65 paired GC and health controls. (SAV)
